# Extra-Articular Lateral Tenodesis for Anterior Cruciate Ligament Deficient Knee: A Case Report

**DOI:** 10.1155/2013/648908

**Published:** 2013-11-28

**Authors:** Diego García-Germán, Pablo Menéndez, Samuel González, Pablo de la Cuadra, Ricardo Rodríguez-Arozena

**Affiliations:** ^1^Department of Orthopaedic Surgery, Hospital Universitario HM de Madrid-Torrelodones, Universidad San Pablo CEU, Torrelodones, 28250 Madrid, Spain; ^2^Department of Orthopaedic Surgery, Servicio de COT, Hospital Universitario de Puerta de Hierro-Majadahonda, C/ Manuel de Falla 1, Majadahonda, 28222 Madrid, Spain; ^3^Department of Orthopaedic Surgery, Hospital Central de la Cruz Roja, 28003 Madrid, Spain

## Abstract

We present the case of an extra-articular lateral tenodesis for an anterior cruciate ligament (ACL) deficient knee. A 46-year-old male patient sustained an ACL graft rupture after a motorcycle accident. He complained of rotational instability and giving-way episodes. His previous graft was fixed by an intra-articular femoral staple that was not possible to remove at the time of the ACL revision. A modified Lemaire procedure was then performed. He gained rotational stability and was able to resume his sporting activities. We believe that isolated extra-articular reconstructions may still have a role in selected indications including moderate-demand patients complaining of rotational instability after ACL graft failure.

## 1. Introduction

ACL revision can be a demanding procedure. Hardware from previous surgeries, tunnel widening, and incorrect tunnel placement, as well as associated injuries, increase complication rates and worse results should be expected compared to primary reconstruction.

Residual positive pivot shift phenomenon after ACL reconstruction has been proposed as one of the key factors affecting patient satisfaction [1]. Rotational instability has been related to the injury and loss of function of the anterolateral structures [2, 3] with the anterolateral ligament receiving increasing interest in recent times [4, 5].

Extra-articular tenodesis were designed to limit internal tibial rotation in ACL deficient knees. Although they are nonanatomic, because they do not reproduce the anterolateral ligament anatomy, they are able to control the pivot shift [6–8]. These techniques were widely abandoned with the introduction of arthroscopic procedures but have showed renewed interest lately in cases where rotational instability is an issue, such as in revision cases [9, 10].

We present the case of an extra-articular lateral tenodesis for an ACL deficient knee with excellent outcome and full patient satisfaction.

## 2. Case Presentation

We present the case of a 46-year-old male patient, who owns a travel agency specialized in skiing and therefore skis over 60 days per season. He had an ACL tear 12 years ago and a Bone-Tendon-Bone autograft ACL reconstruction was performed at the time. He had a very good function until he sustained a motorcycle accident. Since then he complained of rotational instability with giving-way episodes and he was unable to resume his sporting activities.

**Figure 1:**
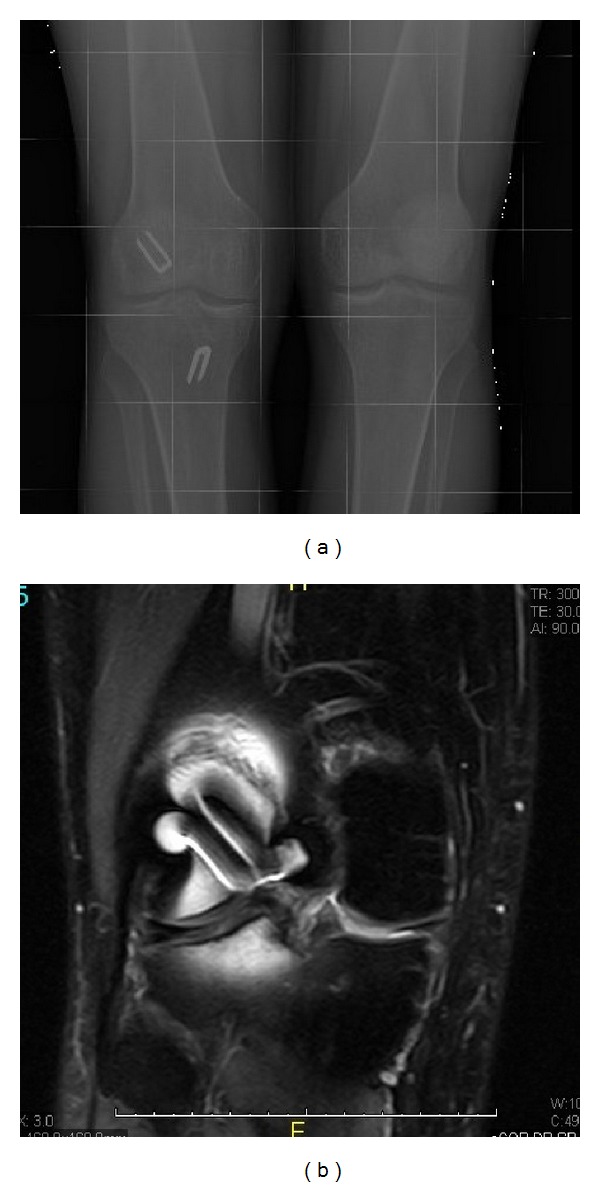
Imaging of the knee showing the presence of intra-articular metal staple and a medial meniscus tear ((a), (b)).

At exploration he presented a positive Lachman test, a positive pivot shift test, and medial joint line tenderness. Plain radiographs revealed staples as the fixation method in his previous ACL reconstruction, with an intra-articular femoral staple (Figure 1(a)). Magnetic resonance imaging (MRI) showed absence of the ACL graft, a medial meniscus tear, and the presence of tibial and femoral metal staples (Figure 1(b)).

The plan was to remove staples and perform an anatomic single bundle ACL reconstruction with autologous quadruple hamstring graft, with a new, more anatomic, femoral tunnel with an outside-in retrograde femoral drilling, which is our standard technique at the present time.

**Figure 2:**
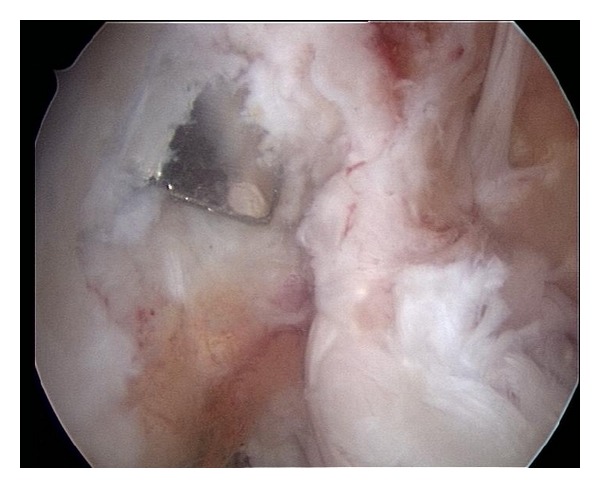
Arthroscopic view of the intercondylar notch showing the presence of the staple. There was space left for an anatomical femoral tunnel but there was concern with the staple affecting graft integrity.

Intra-articular arthroscopic exploration revealed a nonreparable-degenerative tear of the medial meniscus that was resected. Exploration of the intercondylar notch revealed the absence of the previous ACL graft. We were unable to remove the metal staple arthroscopically and the patient had refused an arthrotomy. We did have space to perform an anatomic femoral tunnel, more posterior and distal on the lateral wall, but we were concerned with staple acting as a knife and cutting our graft once placed (Figure 2).

**Figure 3:**
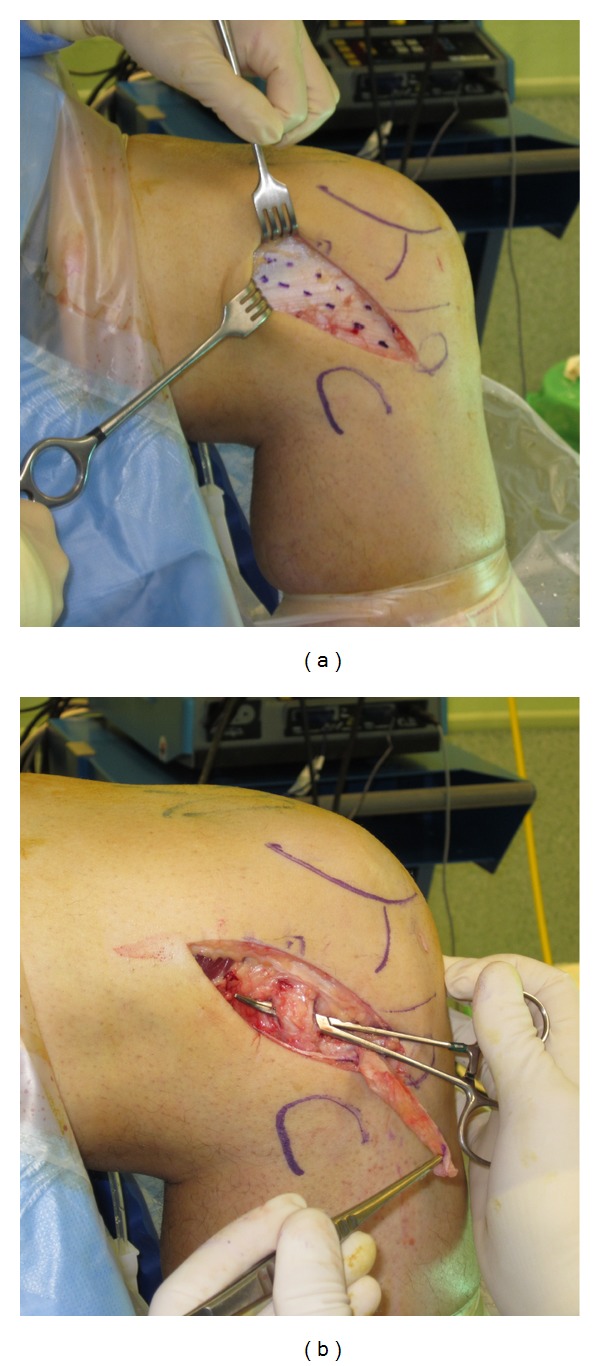
Lateral approach. The graft is designed on the ITB measuring 8–10 cm × 1 cm (a). The space under the LCL is developed, and the graft will be passed under it (b).

We decided to perform an extra-articular tenodesis, by means of a modified Lemaire procedure. An 8 cm long incision was carried out centred over the lateral epicondyle. Dissection was carried down to the iliotibial band (ITB) fascial layer. The graft was designed having 8 to 10 cm in length and 1 cm wide (Figure 3(a)). Distal insertion in Gerdy's tubercule was left in place.

**Figure 4:**
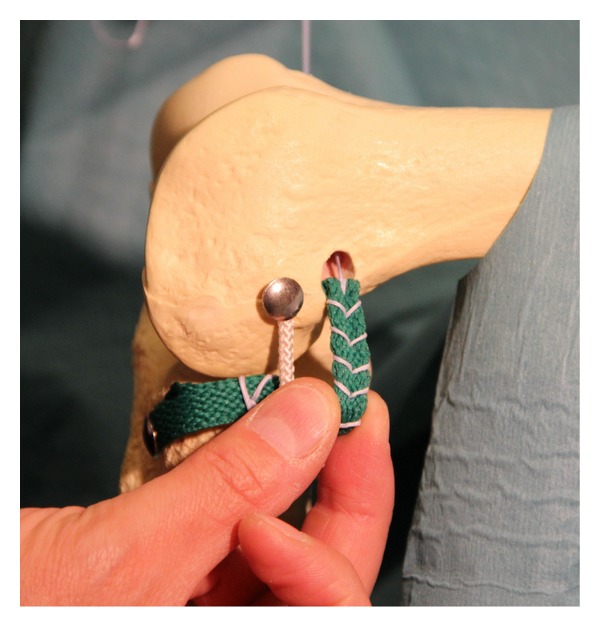
The graft is reinforced with strong, solid-core sutures to avoid graft damage when the interference screw is placed.

The lateral collateral ligament (LCL) was identified and a space under it was developed (Figure 3(b)). The graft was passed under the LCL and the isometric point proximal and posterior to the lateral epicondyle was identified. A guide pin was passed through the distal femur from lateral to medial. The graft was then prepared. We find it important to reinforce the graft with strong, solid-core sutures such as the Fiber-Loop (Arthrex, Naples, FL) to avoid graft damage when the interference screw is placed (Figure 4). We also do this on the tibial side of our standard ACL grafts.

**Figure 5:**
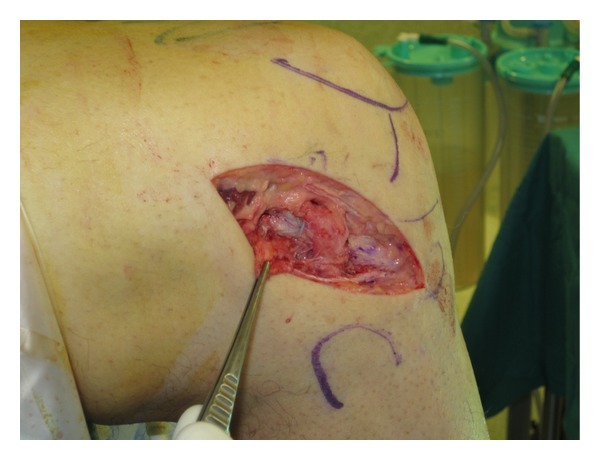
Final image of the tenodesis before closure.

The graft diameter was then measured and a 3 cm deep socket was drilled over the guide pin. The graft was introduced in the socket. Isometry of the graft was checked in range of motion and the role the tenodesis plays in limiting tibial internal rotation could be seen. The graft is secured with an interference screw (Bio-Interference Screw, Arthrex, Naples, FL) (Figure 5).

Postoperative care was slightly faster than we do in standard ACL reconstruction due to the favourable biologic environment of extra-articular tunnel graft healing [11]. The patient presented a completely negative pivot shift and a slightly positive Lachman test with a soft endpoint. At 8-month followup the patient is satisfied with the treatment, feels that his knee is stable, has not had giving-way episodes, and has fully resumed his sporting activities.

## 3. Discussion

The ACL is composed of 2 functionally different bundles, with the anteromedial (AM) controlling sagittal translation and the posterolateral (PL) controlling rotational stability [12]. When performing ACL reconstruction surgery this anatomy should be reproduced to reestablish proper function. The trend has therefore switched from nonanatomic single bundle transtibial reconstruction to more anatomic techniques such as double bundle, anatomic anteromedial portal, or outside-in femoral drilling, whether anterograde or retrograde [13].

Residual positive pivot shift phenomenon after ACL reconstruction has been proposed as one of the key factors affecting patient satisfaction [1]. Rotational instability and the pivot shift phenomenon have been related to the injury and loss of function of the anterolateral structures [2, 3]. The avulsion of these structures during the initial instability episode produces the typical Segond fracture. Although this lesion is not always present, injury to the anterolateral structures always occurs. The study of the anterolateral ligament and its role on knee stability has received increasing interest in recent times [4, 5]. It has been found to be constant in anatomic dissections with a proximal origin just anterior to the popliteus tendon insertion on the femur and a distal insertion on Gerdy's tubercule.

Extra-articular tenodesis were designed to limit internal tibial rotation in ACL deficient knees [6, 13, 14]. Although being nonanatomic, because they do not reproduce the anterolateral ligament anatomy, they are able to control the pivot shift but unable to control anterior tibial translation [15]. They were widely abandoned with the introduction of nonanatomic, transtibial, arthroscopic ACL reconstruction that, on the other hand, is frequently unable to control rotational stability.

Most of these techniques utilize the ITB, leaving the distal insertion in place and either fixing the proximal end to the femur or looping it under the LCL and fixing it back to the tibia. In the MacIntosh technique the graft was sutured proximally to the intermuscular septum [6]. The exact entry point in the femur has not been completely described but the socket should be created slightly proximal and posterior to the proximal origin of the LCL.

The original Lemaire technique used a long graft that was passed through a tunnel in the femur, passed under the LCL, and fixed to the tibia through a tunnel [14]. This can be simplified securing the graft in the femur with an interference screw in a socket. Some authors prefer twisting the graft 180° for further restrain [16]. Some of the new techniques combine intra- and extra-articular reconstruction [6, 9, 17].

There is still debate on the benefit of adding an extra-articular tenodesis to a standard intra-articular ACL reconstruction [9, 10, 18–21]. Good results have been published in recreational skiers over 35 [22]. The recent awareness on the role of the PL bundle and the importance of restoring rotational stability to obtain the expected results could explain the renewed interest in these techniques [6, 7].

We believe that there are some indications for extra-articular lateral tenodesis. It can be done in combination with intra-articular ACL reconstruction in cases of primary or revision ACL reconstruction where rotational instability is important or when there is a rotational instability after a too vertical transtibial ACL graft. As an isolated procedure it could have a role in PL bundle partial ACL rupture and as salvage procedure for complex revision cases.
